# GENDER DIFFERENCES IN ASSOCIATIONS BETWEEN LIVING ARRANGEMENTS AND HEALTH OF OLDER ADULTS IN VIETNAM

**DOI:** 10.1093/geroni/igae098.2729

**Published:** 2024-12-31

**Authors:** Emiko Takagi, Yasuhiko Saito, Nguen Cong Vu

**Affiliations:** San Francisco State University, San Francisco, California, United States; Nihon University, Tokyo, Japan; Institute of Population, Health, and Development, Dong Da, Ha Noi, Vietnam

## Abstract

This study examined gender differences in the variation of physical and psychological well-being measures across four types of living arrangements among older adults in Vietnam. We analyzed the initial wave data of a nationally representative sample of self-reporting adults 60 years and older with at least one living child (n=5,306) from the Longitudinal Study of Health and Aging in Vietnam (LSHAV). The four types of living arrangements in the analysis included: living alone, living with a spouse, living with a spouse, adult children, and/or grandchildren, and living with adult children and/or grandchildren but without a spouse. The well-being measures included physical health indicators regarding the difficulties with Activities of Daily Living (ADL) and Instrumental Activities of Daily Living (IADL), disability status, and self-rated health. Psychological well-being was assessed based on the CESD depression scores and the UCLA 3-item loneliness scores. The descriptive statistics showed that overall, the poorer health conditions tended to be more prevalent in the household where older adults lived with their adult children and/or grandchildren and without a spouse, compared to counterpart types of living arrangements. The regression analyses on well-being measures showed that men who either live alone or live with children or grandchildren but without a spouse were especially more likely to report emotional loneliness. We discuss gender differences in physical and psychological well-being in conjunction with late-life intergenerational living arrangements and life transitions such as widowhood.

